# The relationship between NAFLD and retinol-binding protein 4 - an updated systematic review and meta-analysis

**DOI:** 10.1186/s12944-022-01771-2

**Published:** 2023-01-21

**Authors:** Rui Hu, Xiaoyue Yang, Xiaoyu He, Guangyao Song

**Affiliations:** 1 Department of Internal Medicine, Hebei Medical University, Shijiazhuang, 050017 Hebei People’s Republic of China; 2 Endocrinology Department, Hebei General Hospital, Shijiazhuang, 050051 Hebei People’s Republic of China

**Keywords:** Non-alcoholic fatty liver disease, RBP4 protein, human, meta-analysis, Systematic review

## Abstract

**Purpose:**

Retinol-binding protein 4 (RBP4) has been considered to be related to metabolic related diseases, such as hyperuricemia, obesity, and diabetes mellitus. However, whether nonalcoholic fatty liver disease (NAFLD) is related to RBP4 is unclear. Previous studies on the relationship between NAFLD and RBP4 levels have yielded inconsistent results. Hence, this meta-analysis was aimed to clarify whether circulating RBP4 levels are in relation to the risk of NAFLD.

**Methods:**

A meta-analysis was performed by applying observational studies to evaluate circulating RBP4 levels and NAFLD. Eligible studies published up to September 23, 2022, were searched in Embase, PubMed, and Cochrane databases.

**Results:**

In this study, 17 cross-sectional studies involving 8423 participants were included. Results from a random effects model showed that circulating RBP4 levels were higher in NAFLD patients than non-NAFLD (standardized mean difference (SMD) 0.28; 95% confidence intervals (CI): 0.11–0.46, I^2^: 89.8%). This association was confirmed in the Yellow race. However, no significant association was noted in the Caucasian race. After excluding the morbidly obese Population from the weight loss study (*n* = 2), the results of the comparison remained largely unchanged (SMD 0.28; 95% CI: 0.10–0.47, I^2^: 90.8%). Remarkable publication bias was not found. Although considerable heterogeneity was observed among the studies, no potential sources of heterogeneity were found in the subgroup analysis. Diagnostic methods for NAFLD were determined to be a potential source of statistical heterogeneity in meta-regression.

**Conclusion:**

The findings provide evidence that NAFLD patients exhibit higher levels of circulating RBP4 compared with controls, but high heterogeneity was observed. Thus, a high RBP4 level is probably a potential risk factor for NAFLD. To confirm the causal link between NAFLD and RBP4 level of causality, further prospective cohort studies are needed.

## Introduction

Non-alcoholic fatty liver disease (NAFLD) is a major public health issue impacting about one quarter of the global population [1] and its prevalence is predicted to grow to 56% in the next decade [2]. NAFLD, including simple steatosis (SS) and non-alcoholic steatohepatitis (NASH) that can develop into cirrhosis and hepatocellular carcinoma (HCC) [3, 4], is closely related to metabolic syndrome. Existing research suggests that NAFLD may not only progress to intrahepatic diseases, such as hepatocellular carcinoma, but is also closely related to extrahepatic diseases, such as cardiovascular disease, obesity, diabetes, and hyperuricemia [5]. Hence, treating NAFLD is imperative.

Retinol binding protein 4 (RBP4) is a 21 kDa protein belonging to the Lipocalin family and is the only known particular transporter protein for vitamin A, which regulates the circulating levels of retinol [6]. RBP4 is primarily synthesized and released from the adipose and the liver [7], and it is an adipokine that plays a vital role in growth, vision and metabolic diseases [8]. Free RBP4 has a low molecular weight and is generally bound to transthyretin in a 1:1 ratio in the circulation to keep it from filtration by the glomerulus of the kidney [9].

Till date, the etiology and pathogenesis of NAFLD has not been fully elucidated. Numerous studies have indicated that it involves an intricate interaction among obesity, environmental factors, microbiota changes, and susceptibility gene variants [10–15]. These interactions lead to disturbed lipid homeostasis and excessive accumulation of lipidic substances, such as triglycerides, in hepatocytes [16]. Of these, insulin resistance (IR) is the core mechanism. Serum RBP4 has been reported to be elevated in animal models of IR. In some human studies, a positive correlation has been observed among obese adults between RBP4 levels and fasting glucose and homoeostasis model assessment of insulin resistance (HOMA-IR) [17, 18]. Therefore, NAFLD and RBP4 levels may be correlated. However, there has been inconsistencies regarding the relationship between the two. A previous meta-analysis [19] dating back to 2017 suggests that the two are not correlated, but numerous studies have been published after that. In this research, new studies were included to ensure an updated meta-analysis and systematic review.

## Methods

This meta-analysis is registered on prospero (ID: CRD42022362281).

### Search strategy

**Fig. 1:**
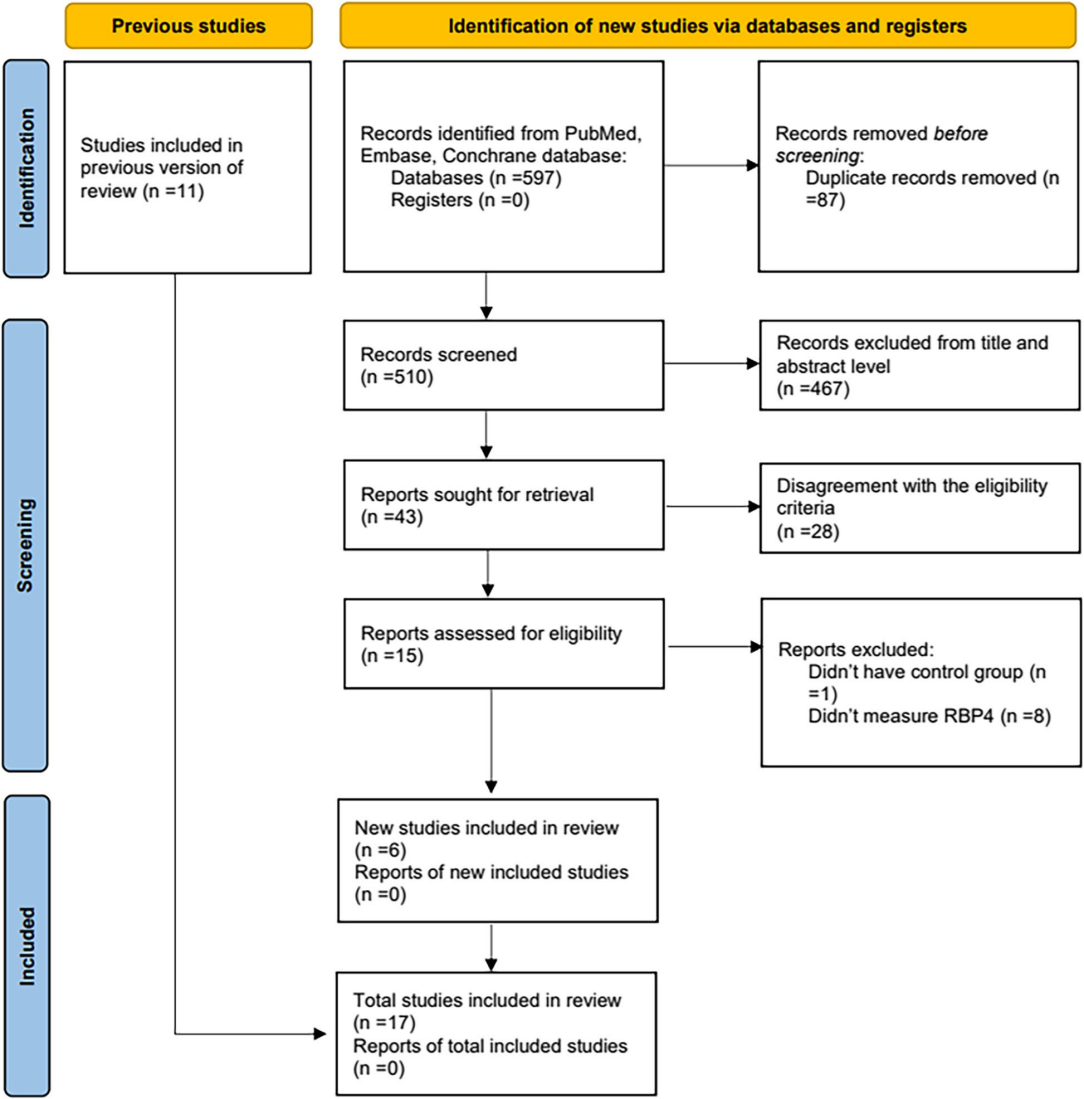
The flow diagram of literature research and study selection

Two independent reviewers (Rui Hu and Xiaoyue Yang) applied the subject terms “non-alcoholic fatty liver disease” and “retinol binding protein 4” and their corresponding free words in the Embase, PubMed, and Cochrane database for studies published before September 23, 2022. Without setting any language restrictions, all potentially eligible studies were included. In addition, the review articles and reference lists in the relevant original studies were examined to search for other potentially eligible researches. Based on the search strategy, all literature collected was independently assessed by two authors (Rui Hu and Xiaoyue Yang). They were both blinded to each other’s records and sought out potentially eligible studies. Any issues raised were addressed through discussion with the fourth author, Guangyao Song. A flow chart depicting the literature selection methodology is given in Fig. 1.

### Inclusion and exclusion criteria

Inclusion criteria: (1) observational researches involving humans; (2) age ≥ 18 years; (3) studies involving measurement of the circulating RBP4 levels in NAFLD and non-NAFLD patients.

Exclusion criteria: Past studies wherein the participants had liver injury due to infectious factors (such as hepatitis B or hepatitis C virus), total parenteral nutrition, alcohol use, drug use, or genetic causes. After excluding these articles, those with insufficient data or those studies that did not include non-NAFLD population were excluded.

### Data extraction

Two researchers (Rui Hu and Xiaoyue Yang) used a specially designed form extracted the independently the necessary data. Any discrepancies were resolved by discussion or by involving a fourth reviewer (Guangyao Song) whenever deemed necessary. The extracted data included the publication year, first author’s name, the design of study, participants’ characteristics (age, the number of females, race, and body mass index [BMI]), NAFLD diagnosis methods, and the technique of measuring RBP4. To sought the missing details, the appropriate person was contacted.

### Quality assessment

Two researchers (Rui Hu and Xiaoyue Yang) assessed the extracted researches. The modified Newcastle–Ottawa Quality Assessment Scale (NOS) [20] was applied to evaluate the study quality. Low quality studies were defined as less than 3 points, moderate quality studies as 4–6 and high-quality studies as 7–9.

### Statistical analyses

Stata14.0 software was applied to conduct all statistical analyses. The standardized mean difference (SMD) was applied to evaluate the study outcomes. In addition, the mean ± standard deviation (SD) was expressed as the normally distributed data. Considering the potential for significant heterogeneity between the enrolled studies, this meta-analysis opted for a more conservative random-effects model. Two-tailed *P*-value was extracted in this meta-analysis.

Using Q and I^2^ statistics, the heterogeneity was evaluated. For Q statistics and I^2^ statistics, *P* < 0.10 and I^2^ < 50% was considered as statistical significance. An I^2^ values of 25% was poor heterogeneity, 50 and 75% represented medium and high heterogeneities, respectively [21].

Through subgroup analyses, the influence of the potential factors on the correlation was assessed, which included ethnicity, the method of diagnosing NAFLD, and the method of measuring the RBP4 levels. In addition, possible sources of heterogeneity were analyzed using meta-regression.

The stability of the outcomes was evaluated for sensitivity analyses by removing individual studies. In addition, Egger’s, Begg’s tests, and funnel plot were applied to assess the publication bias.

## Results

### Literature search

The initial literature search resulted in the identification of 597 articles. After removing 87 duplicates and 467 irrelevant articles, 43 articles remained. After full text screening, 28 articles were excluded because of disagreement with the eligibility criteria listed in Fig. 1. Finally, 17 studies were pooled, all of which were cross-sectional studies.

### Research characteristics

The features of the studies included in this article were shown in Table 1. In brief, 8432 subjects, including 3989 patients with NAFLD and 4443 healthy controls were involved in these 17 studies. Of these, nine studies had a predominantly Caucasian race and eight studies had a predominantly Yellow race. In all studies, 15 studies used enzyme-linked immunosorbent assay to determine RBP4 levels and 2 studies used immunoturbidimetric assay to determine RBP4 levels. Of the 17 studies, 11 studies employed the abdominal ultrasound technique to determine NAFLD, 5 used liver biopsy, and 1 study combined the two methods. Serum RBP4 levels, number of (female) subjects, age, and BMI levels were reported.

**Table 1:** Main characteristics of the studies included in this meta-analysis

References	Country	Study design	Method of RBP4 measurement	Diagnostic methods of NAFLD	Quality Score
Seo(2008) [22]	Korea	Cross-sectional	ELISA	Ultrasound	5
Wu(2008) [23]	China	Cross-sectional	Radioimmunoa-ssay	Ultrasound	6
Kashyap(2009) [24]	USA	Cross-sectional	ELISA	Liver biopsy	7
Koh(2009) [25]	Korea	Cross-sectional	ELISA	Ultrasound	5
Milner(2009) [26]	Australia	Cross-sectional	ELISA	Liver biopsy	5
Schina(2009) [27]	Greece	Cross-sectional	ELISA	Liver biopsy	5
Cengiz(2010) [28]	Turkey	Cross-sectional	ELISA	Ultrasound and liver biopsy	4
Auguet(2013) [29]	Spain	Cross-sectional	ELISA	Liver biopsy	6
Polyzos(2014) [30]	Greece	Cross-sectional	ELISA	Liver biopsy	5
Suh(2014) [31]	Korea	Cross-sectional	ELISA	Ultrasound	6
Chen(2017) [32]	China	Cross-sectional	ELISA	Ultrasound	4
Cai(2018) [33]	China	Cross-sectional	ELISA	Ultrasound	5
Ashmawy(2019) [34]	Egypt	Cross-sectional	ELISA	Ultrasound	5
Wang(2020) [35]	China	Cross-sectional	ELISA	Ultrasound	5
Ikizek (2020) [36]	Turkey	Cross-sectional	ELISA	Ultrasound	4
Hassan(2021) [37]	Iraq	Cross-sectional	ELISA	Ultrasound	3
Zhang (2022) [38]	China	Cross-sectional	ELISA	Ultrasound	4

### Quality evaluation

A modified NOS scale was used to assess the quality of the included studies. As presented in Table 1, of all studies, 15 were evaluated to be of moderate quality, 1 of high quality, and 1 of low quality.

### Statistical results

**Fig. 2:**
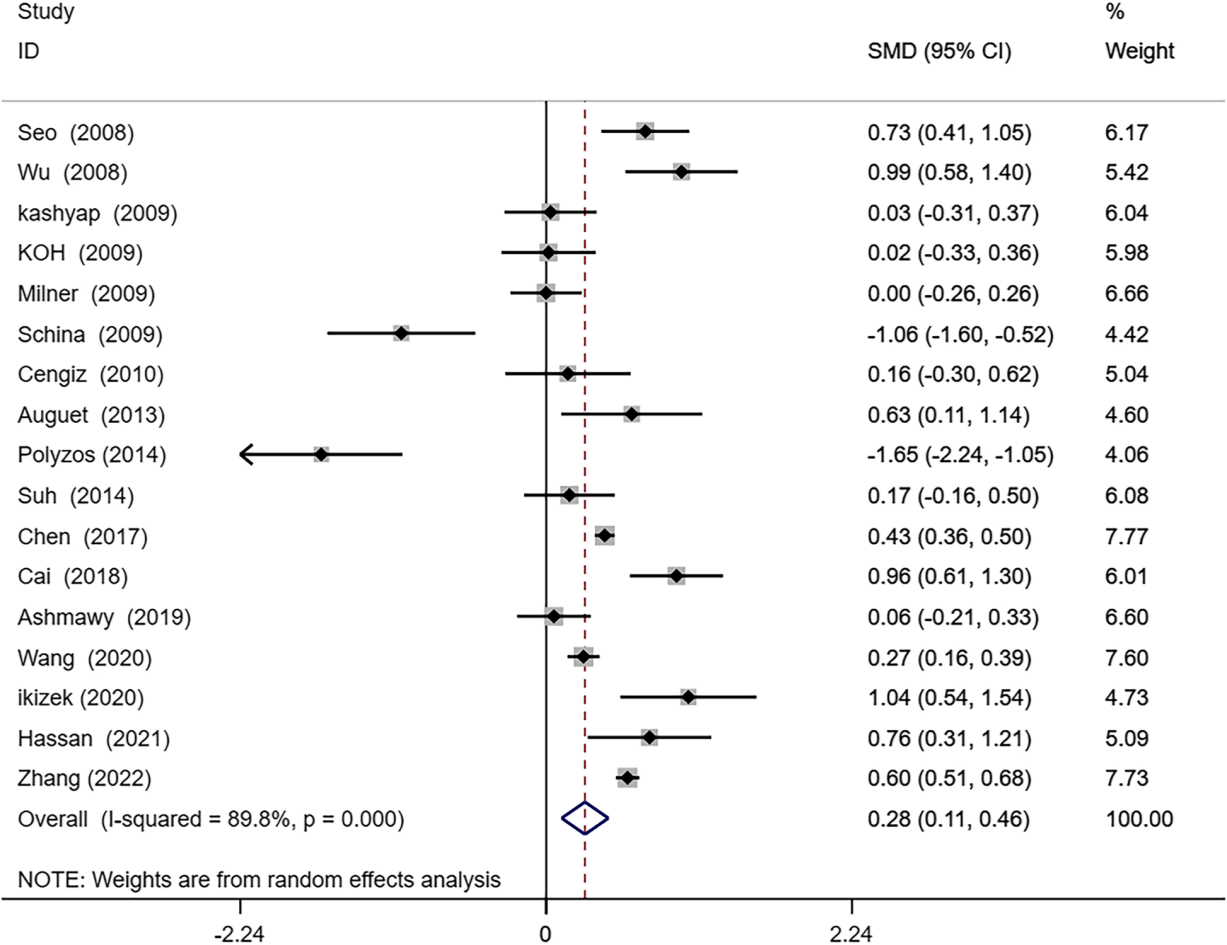
Forest plot of circulating retinol-binding protein 4(RBP4) levels between nonalcoholic fatty liver disease(NAFLD) and the control group

A random effects meta-analysis was conducted on the 17 studies included in this article. The results showed that serum RBP4 levels was higher in NAFLD patients than non-NAFLD (SMD 0.28; 95% CI: 0.11–0.46, I^2^: 89.8%, *P* = 0.001) (Fig. 2).

### Subgroup analysis

**Fig. 3:**
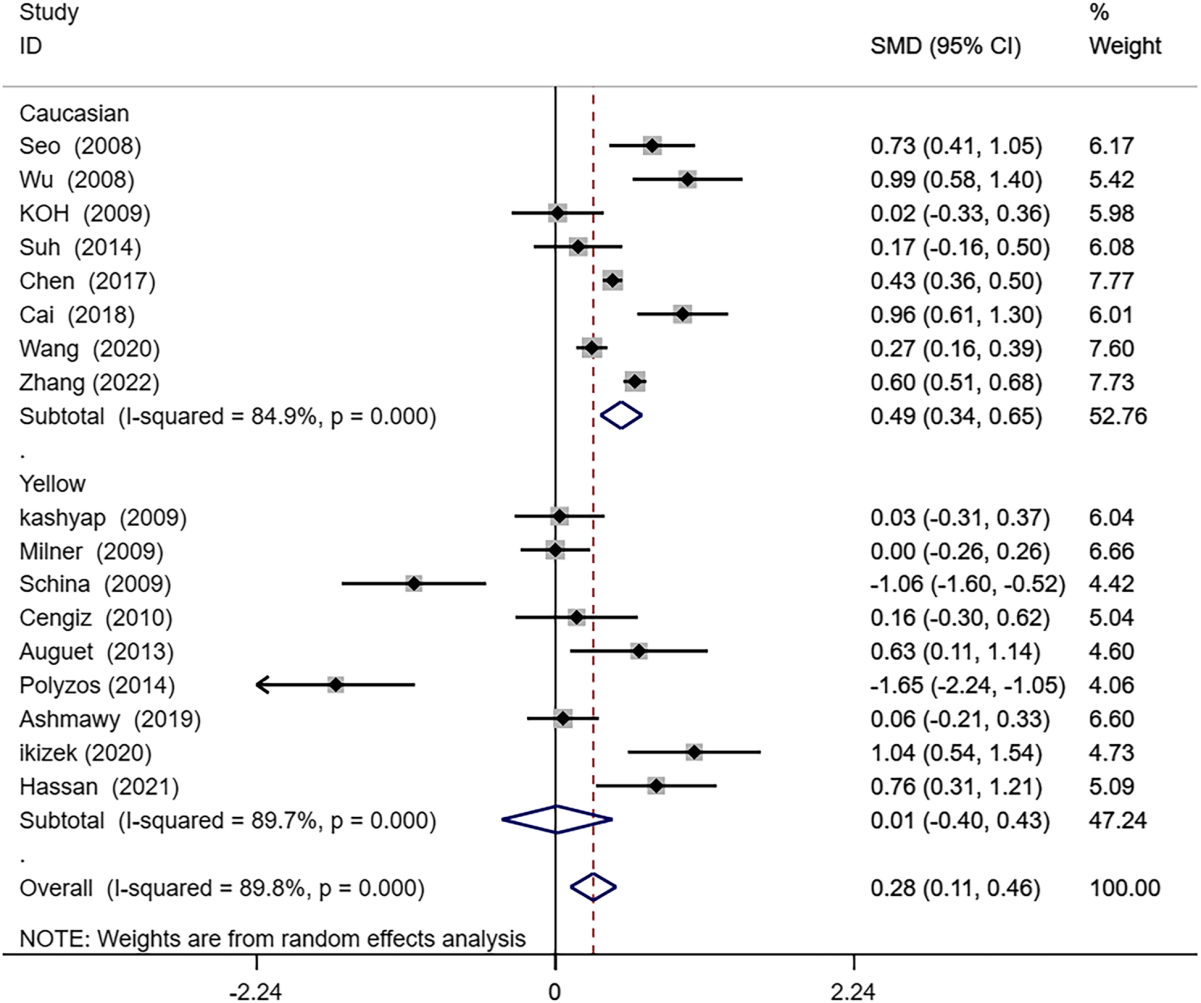
Subgroup analysis by ethnicity

To examine whether differences in ethnicities (Caucasian, Yellow race) could be the source of the high subgroup analysis was conducted according to the different ethnicities included in the study. As shown in Fig. 3, Yellow ethnic patients with NAFLD presented notably higher circulating RBP4 levels than non-NAFLD (SMD0.49; 95% CI: 0.34–0.65; *P* = 0.001). However, circulating RBP4 levels in Caucasian NAFLD patients were not statistically different from those of healthy controls (SMD0.01; 95% CI. -0.40–0.43; *P* = 0.949). Nevertheless, there remained a high level of heterogeneity in the comparison between Yellow (I^2^ = 84.9%; *P* = 0.000) and Caucasian (I^2^ = 89.7%; *P* = 0.000) populations.

**Fig. 4:**
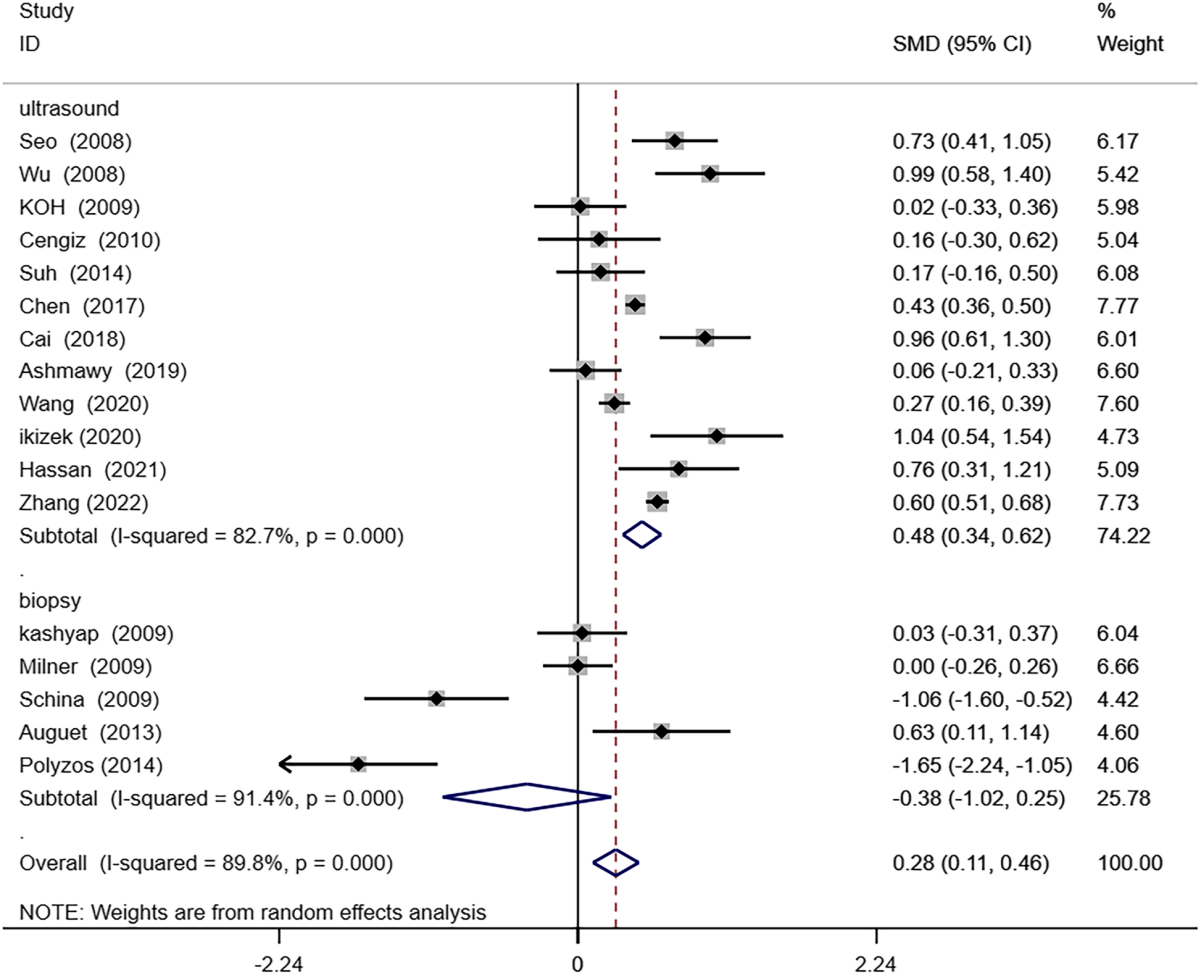
Subgroup analysis by diagnostic method of nonalcoholic fatty liver disease (NAFLD)

Next, based on the diagnostic method of NAFLD, subgroup analysis was performed. In the subgroup with abdominal ultrasound as the diagnostic method, NAFLD patients had remarkably higher circulating RBP4 levels than those non-NAFLD (SMD 0.48; 95% CI. 0.34–0.65; *P* = 0.000). However, in the subgroup with liver biopsy as a diagnostic method, patients with NAFLD had no statistically significant difference in circulating RBP4 levels compared to non-NAFLD (SMD -0.38; 95% CI: − 1.02–0.25; *P* = 0.235). These results suggest that the circulating RBP4 was higher in patients with abdominal ultrasound as a diagnostic method than in controls. (Fig. 4).

### Sensitivity analysis and meta-regression

**Fig. 5:**
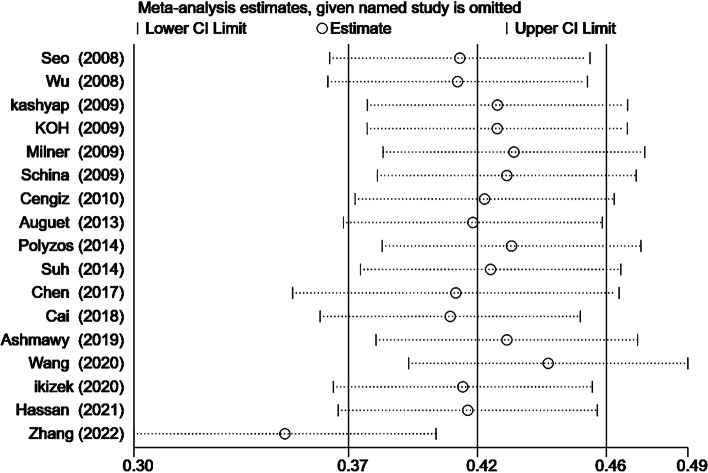
Sensitivity analysis plot

**Fig. 6:**
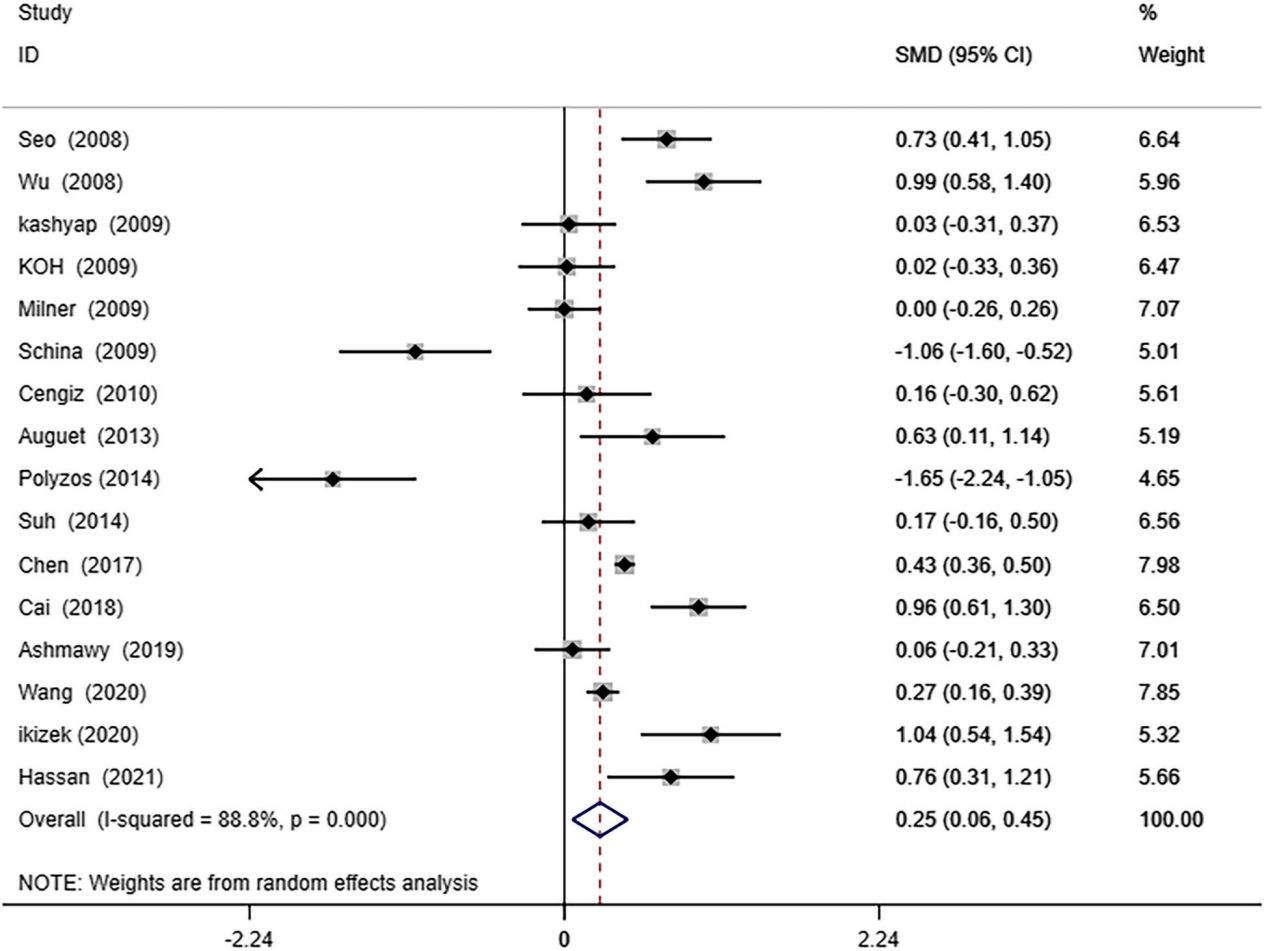
Forest plot of circulating retinol-binding protein 4(RBP4) levels between nonalcoholic fatty liver disease(NAFLD) and the control group after excluding studies with remarkable effect on the deviations

**Fig. 7:**
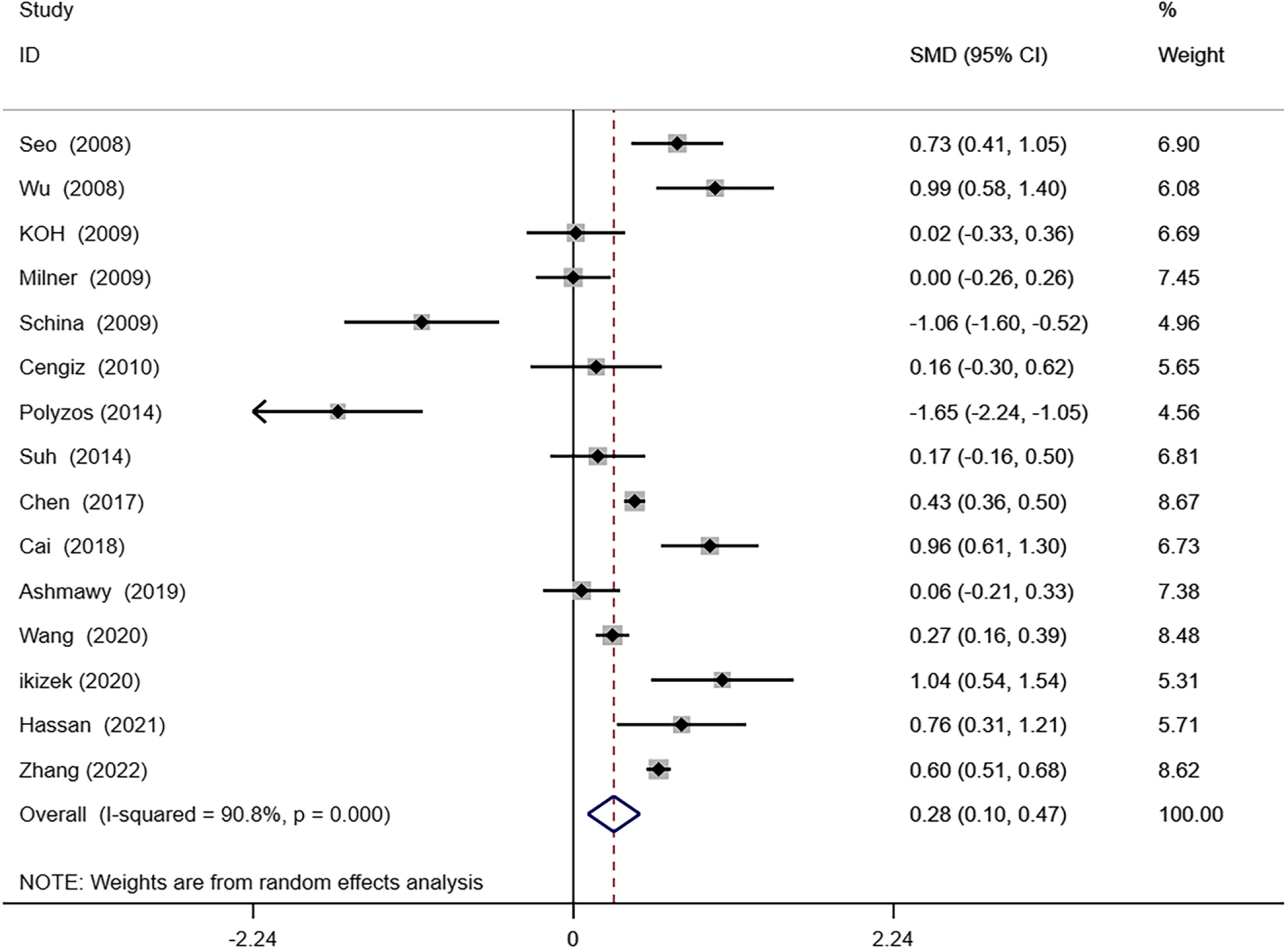
Forest plot of circulating retinol-binding protein 4(RBP4) levels between nonalcoholic fatty liver disease(NAFLD) and the control group after excluding the weight loss studies

One study was found to exert a remarkable effect on the deviations in RBP4 levels between patients with NAFLD and without in sensitivity analysis (Fig. 5). Hence, this article was removed, and a new meta-analysis of the remaining 16 studies was conducted. The results indicated that NAFLD patients had higher circulating RBP4 levels than non-NAFLD (SMD 0.25; 95% CI: 0.06–0.45, I^2^: 88.8%, *P* = 0.011) (Fig. 6). Furthermore, after excluding the weight loss study (*n* = 2), only a small change was noted in the comparison between NAFLD patients and without NAFLD (SMD 0.42; 95% CI: 0.38–0.47; *P* = 0.000) (Fig. 7).

In the meta-regression analysis (Table 2), the diagnostic method of NAFLD may be a potential source of high heterogeneity (univariate meta-regression: *P* = 0.002; multivariate meta-regression: *P* = 0.024).

**Table 2:** Meta-regression of the circulating RBP4 levels and NAFLD

Covariates	No. Groups	Coefficient	Standard error	t	P	95%CI	
Univariate meta-regression analysis							
RBP4 measurement	17	0.598	0.477	1.25	0.210	−0.337	1.533
Quality scores	17	−0.110	0.174	−0.64	0.525	−0.451	0.230
Number of females	17	0.000	0.001	0.45	0.655	−0.001	0.002
BMI	16	−0.011	0.023	−0.49	0.625	−0.057	−0.034
NAFLD diagnosis	17	−0.869	0.286	− 3.04	0.002	−1.430	−0.308
Age	16	−0.015	0.032	−0.46	0.648	−0.078	0.049
Ethnicity	17	−0.502	0.300	−1.67	0.094	−1.091	0.086
Multivariate meta-regression analysis							
NAFLD diagnosis	17	−0.854	0.377	−2.27	0.024	−1.593	−0.115
Ethnicity	17	−0.028	0.337	−0.08	0.934	−0.689	0.886

*CI* Confidence interval, *BMI* Body mass index, *NAFLD* Non-alcoholic fatty liver disease, *RBP4* Retinol-binding protein 4

### Publication bias

**Fig. 8:**
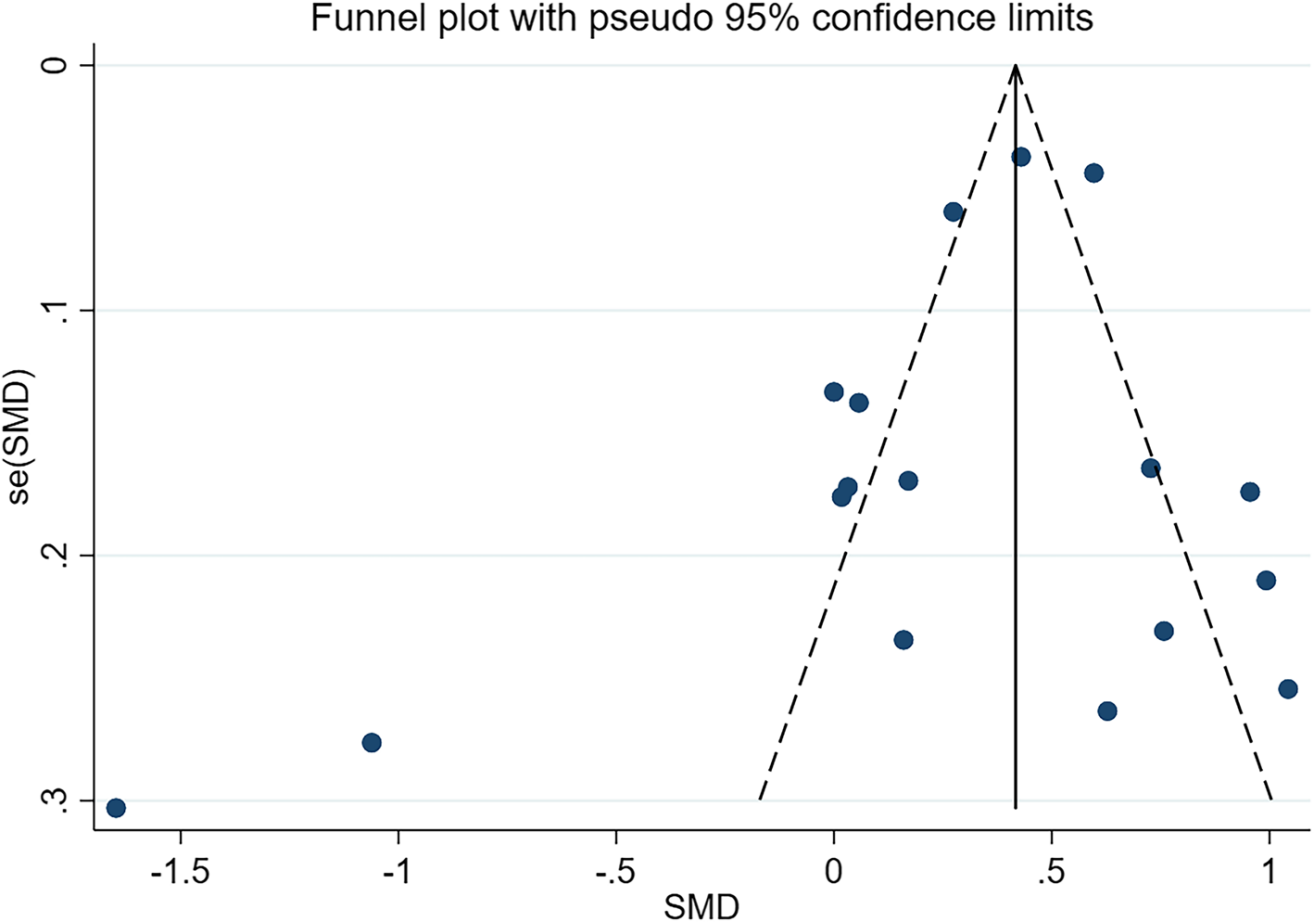
Funnel plot

**Fig. 9:**
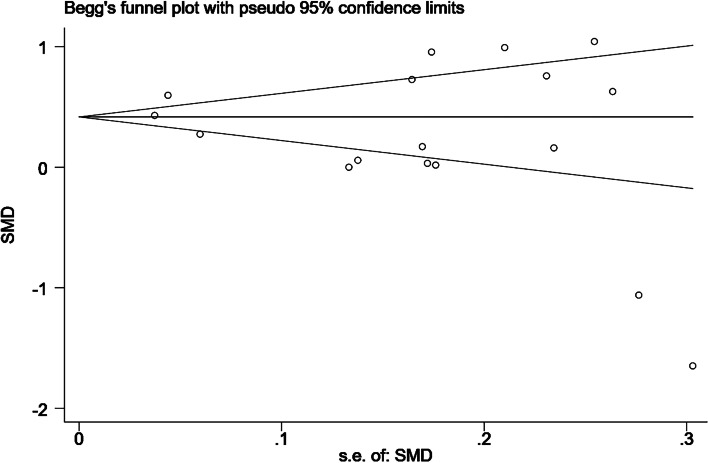
Begg's test

**Fig. 10:**
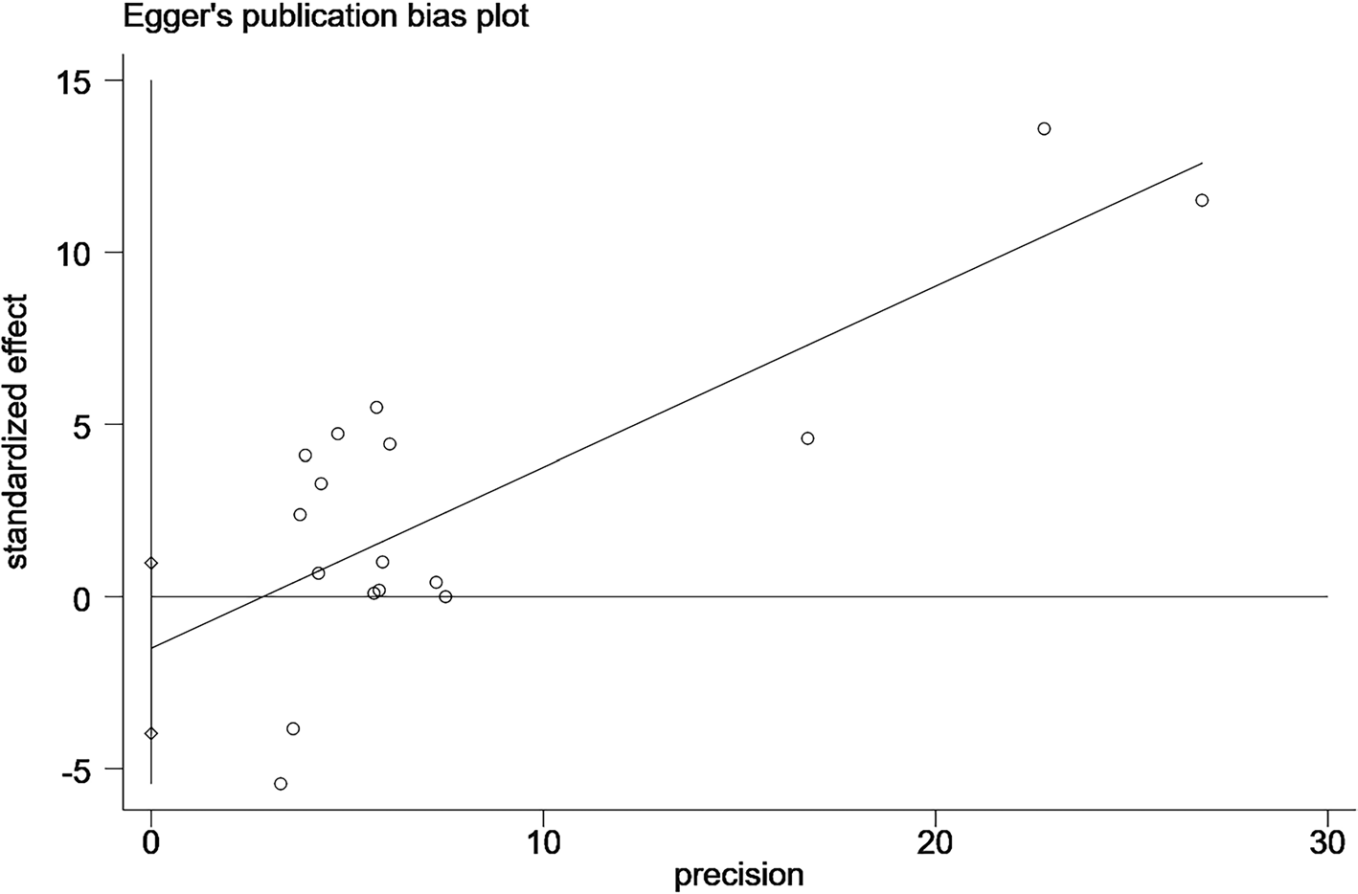
Egger's test

Given the subjective nature of funnel plots, we performed an analysis of publication bias using the Egger test and confirmed the absence of publication bias (*P* = 0.217) (Figs. 8, 9, 10).

## Discussion

In this meta-analysis, higher levels of circulating RBP4 were found in NAFLD patients compared to non-NAFLD, although heterogeneity between the combined studies was significant. The findings of a previous meta-analysis (SMD = 0.08, 95% CI: − 0.21 to 0.38, *P* > 0.05) disagree with the results of this one [19]. However, the broader population and the higher number of participants in the present study might have improved the consistency of the results. Several studies have investigated the relationship between RBP4 levels and NAFLD but have reported varying results. In 3389 individuals with and without NAFLD, a positive connection between circulating RBP4 levels and NAFLD development was observed after 3.09 years of follow-up [Odds ratio (95% CI) for the development of NAFLD (three-digit: 2.01, (1.33, 3.04)] [35]. In contrast, another research showed that circulating RBP4 levels had no relation with the development of NAFLD, as confirmed with liver biopsy [26]. Thus, the results are conflicting, which could be attributed to the differences in diagnostic methods (e.g. abdominal ultrasound and liver biopsy), age, sex composition and sample size. Furthermore, studies that reported serum RBP4 to be an independent risk factor for NAFLD were all conducted in Yellow racial populations, whereas those in Western countries mostly reached opposite conclusions, which may be related to ethnic variability. Therefore, the relationship between RBP4 levels and NAFLD and its severity must be elucidated in larger clinical studies.

The mechanism by which RBP4 causes NAFLD progression remains unclear. Liu [39] found via animal experiments that the overexpression of RBP4 could aggravate liver mitochondrial dysfunction and promote steatosis in mice by impairing the oxidative capacity of the liver mitochondria, thereby suggesting that RBP4 is a possible target for liver fat accumulation. Moreover, IR may be one of the critical mechanisms by which NAFLD is associated with RBP4. As an important mechanism of NAFLD occurrence, IR could result in the impairment of the ability of adipocytes to store fat, leading to the release of free fatty acids into the bloodstream, which promotes hepatic lipid deposition and exacerbating the development of NAFLD [40]. However, the relationship between RBP4 and IR is controversial, and the first strong link between RBP4 and IR was demonstrated by Yang [7]. The researcher discovered that circulating RBP4 levels were correlated with the degree of IR in patients, including obese patients, impaired glucose tolerance patients, and type 2 diabetics as well in normal participants with a family history of these diseases. A subsequent study showed that in obese and type 2 diabetic patients, circulating RBP4 level was positively related to metabolic syndrome and IR [41]. Additionally, recent studies have indicated that circulating RBP4 levels were related to IR in people with hyperuricemia [42]. Other studies [27, 43] have arrived at the opposite conclusion, but in these studies, HOMA-IR was used to assess IR rather than the orthoglucose clamp assay, which is the golden standard for assessment. Furthermore, RBP4 is also strongly related to inflammation, and the progression of inflammation in the liver is a factor that influences the advancement of NAFLD because it exacerbates the transformation of simple steatosis into steatohepatitis [16].

In this meta-analysis, high heterogeneity between studies was found when performing subgroup analyses. Moreover, meta-regression suggested that the diagnostic method of NAFLD may be a potential source of heterogeneity. After subgroup analysis based on the diagnostic method of NAFLD, the heterogeneity reduced slightly. Nonetheless, there were no remarkable differences in NOS scores, patient age, sex, measurement method, or diagnostic methods for NAFLD before and after the exclusion of outliers; hence, these results are robust. During the measurement of RBP4 level, only some studies grouped the values according to the severity of NAFLD, Zhou [19] did not find differences in RBP4 levels among the three groups based on a two-by-two comparison of SS, NASH, and control groups. Furthermore, there have been no new studies have since then to compare the RBP4 levels between the three groups. Therefore, the severity of NAFLD could possibly affect the final results.

Genetic variants in the I148Met allele of *PNPLA3* and the 167Lys allele of *TM6SF2* have been shown to be strongly linked to the increase in hepatic fat deposition and the development of cirrhosis and hepatocellular carcinoma [44]. Additionally, some studies [45, 46] have shown a correlation between serum RBP4 levels and renal insufficiency and that NAFLD is associated with reduced glomerular filtration rate and/or microalbuminuria [47]. Only a few of the included studies specified the glomerular filtration rate and urinary microalbumin values; some studies included these with renal insufficiency as an exclusion criterion, but some studies were not rigorously controlled. Furthermore, one study [34] showed that circulating RBP4 levels were lower in premenopausal NAFLD women than in postmenopausal NAFLD women. This finding implies that serum RBP4 levels could be affected by the menopausal status and that estrogen deficiency may adjust the circulating RBP4 levels downward.

### Strengths and limitations

The current meta-analysis including 17 clinical researches has a larger sample size than previous meta-analyses and includes literature from a wider region, including Asia, Europe, and Africa [33–35, 37, 38].

However, some limitations are present. First, all the included studies were cross-sectional studies, and the causal relationship between RBP4 levels and NAFLD could not be established. Second, although the indicators of circulating RBP4 levels were converted to the same units, the values varied from one study to the other. For patients with NAFLD, the highest RBP4 level was 94.6 μg/mL [36], whereas the lowest was only 0.69 μg/mL [30]. This variation may be related to the differences in the assay methods and experimental kits. Third, the results showed some heterogeneity, which could be ascribed to diet, varying proportions of the diabetic population, etc. In addition, all studies were subject to bias, and although some studies had controlled for factors such as age and sex, many unexpected factors may still exist. Studies have shown that the diagnostic methods for NAFLD may be a source of heterogeneity, but the application of liver biopsy as a diagnostic method for NAFLD has been less well studied, which inevitably has certain deviations. Fourth, although RBP4 belongs to the lipid family and is closely related to IR, HOMA-IR has not been covered in most articles [24, 36–38]. Thus, it is impossible to perform reasonable subgroup analysis and meta-regression based on HOMA-IR. Finally, according to the subgroup analysis, the circulating RBP4 levels in the Yellow racial NAFLD population were different from those in the normal population of the same race. However, this discrepancy was not observed in the White race, which might be because of the inclusion of articles mainly from the Yellow race. Therefore, whether the results would be applicable to other ethnicities is uncertain. More data on other ethnicities are, hence, needed to draw comprehensive conclusions.

## Conclusions

Patients with NAFLD exhibited higher levels of RBP4 than healthy controls. Hence, RBP4 may be an emerging noninvasive biomarker for NAFLD. In addition, RBP4-targeting agents can be considered for the treatment of this disease.

## Data Availability

Within the article and its supplementary files.
